# Empowering people with acquired brain injury to master their well-being: a thematic analysis of participant experience of an 8-week positive psychotherapy group

**DOI:** 10.1080/17482631.2025.2595847

**Published:** 2025-12-10

**Authors:** Kelly Davies, Zoe Fisher, Andrew Kemp

**Affiliations:** a School of Psychology, Faculty of Medicine, Health & Life Science, Swansea University, Swansea, United Kingdom; b Regional Neuropsychology and Community Brain Injury Service, NHS Wales, Morriston Hospital, Swansea, United Kingdom; c Health and Well-being Academy, Faculty of Medicine, Health & Life Science, Swansea University, Swansea, United Kingdom

**Keywords:** Well-being, patient experience, positive psychotherapy, thematic analysis, neurorehabilitation, psychological boosting, acquired brain injury, improved outcomes

## Abstract

**Purpose:**

Despite lifelong challenges, people with Acquired Brain Injury (ABI) can live meaningful and fulfilling lives. Advancements in well-being science and positive psychology (PP), have shifted focus from reducing illbeing to fostering promoting, defined as striving towards excellence based on one’s unique potential. These developments make well-being an achievable goal for those with chronic conditions. Whilst emerging evidence suggests that PP techniques can enhance well-being in ABI populations, wide-spread application remains limited and unmet psychological and social needs continue. This study explored the feasibility and well-being impact of a psychosocial intervention, across three neurorehabilitation centers in Wales.

**Method:**

This qualitative study presents qualitative findings relating to participant experience of an 8-week group-based positive psychosocial intervention, collected during a from a mixed-methods randomised controlled, mixed-methods feasibility study. Twenty participants from the three neurorehabilitation centers participated in this study. Semi-structured focus groups were analysed using reflexive thematic analysis, combining inductive and deductive coding, to explore meanings.

**Analysis:**

Five themes reflected participant views on the feasibility of the intervention and three on its impact. Participants valued the holistic design, structured psychoeducation, and emphasis on building psychological resources. These findings demonstrate that well-being can be actively enhanced in people with ABI through structured group-based PP interventions. Participants described psychological ‘boosts’ and gains in motivation, self-efficacy, and the capacity to sustain well-being.

**Conclusion:**

These insights provide a strong foundation and practical guidance for a full-scale RCT and future implementation. By translating theory into real-world applications, this study highlights the potential of modern PP-informed approaches to address unmet needs and improve outcomes in individuals with ABI.

## Introduction

Individuals with ABI have experienced an injury to the brain after birth which can either be traumatic, due to an external force, or non-traumatic, caused by internal factors such as stroke (Vaghela et al., 2023). Around 1.3 million people live with an ABI in the UK, and their lives are affected by a range of physical, cognitive, and emotional sequelae (Goldman et al., 2022).

### Neuropsychological sequalae of ABI

The physical effects include sleep disturbances, fatigue, chronic pain, and a range of motor, co-ordination, and sensory impairments (Goldman et al., 2022). Commonly affected cognitive domains include higher-order functions such as processing speed, memory, attention, and executive function (Wood & Worthington, 2017). Additionally, individuals may experience more localised deficits, depending on the location of the injury such as language impairments (e.g., aphasia) or selective memory disorders (e.g., amnesia) (Goldman et al., 2022). Emotional and neurobehavioural difficulties following ABI include personality changes and mental health challenges (Goldman et al., 2022). Social communication deficits can also affect a person’s ability to interact with others, leading to social isolation (Douglas, 2007; Salas et al., 2018). The net effects can be pervasive and hidden with a substantial proportion of people affected unable to return to work (Van Velzen et al., 2009). ABI-related costs in the UK equate to approximately 10% of the NHS budget, making neurorehabilitation one of the most cost-effective interventions available (Parsonage, 2016).

### Ongoing challenges in Rehabilitation for ABI

It has long been recognised that effective neurorehabilitation requires a comprehensive approach to cater to the breadth of severity and nature of the neuropsychological sequelae (Ben-Yishay, 1996). However, adequate provision remains challenged with psychological needs often reported as unmet (Norman et al., 2023) There is a general consensus amongst researchers and clinicians that successful neuropsychological rehabilitation needs to focus on optimising well-being across physical, cognitive, psychological, and social domains, to help people with ABI live fulfilling and meaningful lives (Wilson & Betteridge, 2019). While policies promote the need for a holistic person-centred approach to enable people with ABI to self-manage their well-being, this does not translate into practice (Government of Wales, 2022a; Odumuyiwa et al., 2019). Acknowledging that returning to a pre-injury position is often not attainable, it is crucial to explore what well-being looks like in the context of living with ABI (Wilson & Betteridge, 2019).

### Well-being for people living with ABI

Well-being is no longer considered merely the absence of ill-being (Anderson, 1995; Kemp & Fisher, 2022). Recent developments in well-being theory, including advances in Positive Psychology (PP), have been integral to its application to people with acquired brain injury (ABI). Firstly, the shift, through PP’s first wave, from viewing well-being as the absence of ill-being to its second-wave; understanding it as a multifaceted measure of human flourishing has highlighted the potential to simultaneously enhance well-being while reducing/managing negative symptoms (Wong, 2011). This is particularly relevant for people living with ABI, who often face life-long challenges. For example, the inability to return to work or previous hobbies can negatively impact one’s sense of purpose (Van Velzen et al., 2009), while social communication difficulties can adversely affect relationships (Douglas, 2007; Salas et al., 2018). To optimise well-being, it must be recognised that progress is likely to be non-linear, encompassing both positive and negative emotions and experiences (Gracey et al., 2009). Secondly, through PP’s third wave, contemporary perspectives extended well-being beyond the individual self, recognising the important role of context - a crucial consideration for people living with ABI given its long-term nature, breadth of sequelae, and impacts on individuals, families, and communities (Salas et al., 2021). More recent well-being theories, such as Kemp and Fisher (2022) GENIAL framework and Lomas et al. (2024) Sustainable Well-being model, have consolidated and extended earlier theories to capture the mind–body connection and socio-contextual factors including community, and nature. These frameworks conceptualise well-being as a dynamic system linking self, others, and nature, emphasising the interdependence of personal, social, and ecological wellbeing. Such holistic perspectives provide a valuable foundation for developing interventions that build well-being among people living with ABI (Cullen et al., 2018; Kemp & Fisher, 2022).

### Practical application for building well-being in people with ABI

Research has linked key constructs in PP, such as hope (Bellon et al., 2022), resilience (Bertisch et al., 2014) and living in line with one’s values (Baseotto et al., 2022) to improved quality of life and successful rehabilitation outcomes for people with ABI. These links indicate the potential to build well-being in people with ABI through PP interventions that aim to promote positive behaviours, feelings, or thoughts (Schueller et al., 2014) for example character strengths (Schutte & Malouff, 2019) and gratitude practices (Davis et al., 2016). Research has shown that PP interventions are equivalent to traditional therapies, such as cognitive behavioural therapy (CBT), in improving mental health (Chaves et al., 2017) and more effective in enhancing well-being (Asgharipoor et al., 2012).

Successful adaptation to enhance well-being in people with ABI requires extending interventions to encompass second-wave PP techniques, such as embracing negative experiences and managing difficult emotions (Cullen et al., 2018; Whiting et al., 2020). Research has shown that combining therapy with psychoeducation lays a foundation for knowledge and understanding to empower individuals to make impactful choices to promote their own well-being (Anderson, 1995; Fabian & Pykett, 2022; Hunter, 2020). This presents a valuable opportunity to support people with ABI in self-identifying and employing well-being strategies addressing the recognised gap for tailoring interventions to meet individual needs (Odumuyiwa et al., 2019). While these interventions demonstrated the potential to enhance well-being by combining therapy with psychoeducation (Cullen et al., 2018; Graham et al., 2015), their one-to-one delivery method constrained opportunities for social interaction and mutual support that are often fostered in group contexts (Holt-Lunstad et al., 2010). Connecting with others through shared experience has proven instrumental in supporting people with ABI to adjust to a new sense of self, a critical step in rehabilitation (Salas et al., 2018; Vaghela et al., 2023). Given the strong link between ABI and social isolation (Goldman et al., 2022) offering interventions in a group setting provides a significant opportunity to build well-being through social connection given its key mediating role (Haslam et al., 2008). Evidence from peer mentoring and activity-based programmes shows that shared experiences foster belonging, reduce loneliness, and enhance well-being (Aterman et al., 2023; Gibbs et al., 2022; Payne et al., 2020).

Research into the effectiveness of well-being interventions offering both psychoeducation and opportunities for social connection for people with ABI is limited. Research and theory indicate that both play a role in optimising well-being: psychoeducation equips people with the knowledge to improve their well-being, while social connection provides a route to self-acceptance through to similar others (Fabian & Pykett, 2022; Hawley et al., 2022; Salas et al., 2018).

Recently published US studies have indicated improved quality of life for people with ABI who attended group-based interventions combining psychoeducation, mindfulness, and yoga (Callahan et al., 2023; Donnelly et al., 2020). Tulip et al. (2020) study of group psychotherapy, based on the original GENIAL framework (Kemp & Fisher, 2022), demonstrated the potential of such interventions to enhance well-being by promoting positive emotions, social opportunity, and empowerment. Advancing beyond this, our intervention is explicitly designed around an interconnected systems perspective, treating self connection, social connection, and nature connection as interdependent targets that can amplify one another. The absence of widespread adoption of these types of interventions offers a potential explanation for why people with ABI continue to report unmet psychological needs (Odumuyiwa et al., 2019) aligning with criticism of failure to translate research findings into practice (Grimshaw et al., 2012). To support this effort research evidence is needed to examine participant exeperiene relating to taking part in holistic wellbeing interventions guided by latest research and theory (Gimigliano & Negrini, 2017).

### Present study

As part of a wider mixed-methods RCT feasibility study (Fisher et al., 2024), the present study explored the participant experience of a group-based positive psychotherapy intervention designed to encompass the latest developments in well-being science, focused on re-establishing connections to self, others and nature (Kemp & Fisher, 2022). The intervention integrated aspects of second- and third-wave PP, such as navigating ups and downs, living with difficult emotions, connection to others, and the natural environment (Kemp & Fisher, 2022) tailored to the context of people living with ABI. Co-facilitated by peer mentors, this group-based intervention provided psychosocial education along with reconnecting people to self and others. Recognising the important contribution of qualitative research to feasibility studies (O’Cathain et al., 2015), the aim of the study was two-fold: to understand participants’ perspectives on the feasibility of the intervention as it is offered (Fisher et al., 2024) and to explore the impact of group-based psychosocial intervention in building well-being for people with ABI.

## Method

### Ethics

This study was part of a mixed-methods RCT feasibility study that received full NHS ethical approval from the Wales Research Ethics Committee [IRAS project ID: 271,251, REC reference: 19/WA/0336] (Fisher et al., 2024).

### Design

The RCT feasibility study (Fisher et al., 2024) entailed conducting an 8-week group-based positive psychotherapy intervention (PP intervention) for people with ABI, with the primary aim of ascertaining feasibility and conditions for a fully powered study. Participants were allocated into one of two groups: the control group, who had treatment as usual (TAU), and the PP intervention group. This qualitative component had two aims: to understand participants’ perspectives about the feasibility of conducting a full-scale RCT (Fisher et al., 2024) and to explore the impact of group-based psychosocial intervention in building well-being for people with ABI. Reflexive thematic analysis (RTA) was used to explore, interpret, and draw patterns of meaning from the experiences of multiple participants while embracing the insights generated from the perspective of the researchers (Braun & Clarke, 2021). A critical realist approach was adopted as it recognises that wellbeing and has real effects on people’s lives, yet can only be accessed through the accounts of individuals. This position allowed us to value participants’ and mentors’ narratives as situated interpretations of reality, while also connecting them to wider well-being theory and feasibility criteria. In this way, critical realism enabled us to acknowledge both the reality of well-being experiences and the subjective, contextual ways they are understood and expressed (Braun, 2022; Pilgrim, 2019). The flexibility of RTA enabled both aims of the study to be achieved by tailoring the orientation of the analysis, which adopted a more inductive orientation to understand their experience of the study in terms of its feasibility, and a more deductive approach to interpret participant experience in relation to its the impact of the intervention on well-being (Braun & Clarke, 2021).

#### Reflexivity

Qualitative analysis was conducted by the first author, KD, whose perspective was shaped by both personal experiences of close family and friends affected by brain injury and an academic interest in holistic well-being theory and practice. While new to research on brain injury rehabilitation and well-being, this fresh perspective allowed for an open and exploratory approach to participant experiences. Colleagues with expertise in clinical neurorehabilitation and well-being research have provided alternative viewpoints, contributing to a broader interpretation of the data. Reflexive Thematic Analysis (RTA) facilitates the integration of these multiple perspectives, enriching the analysis and enhancing the depth of interpretation.

### Participants

As part of the RCT, participants with a confirmed diagnosis of ABI were selected using a purposive sampling approach across three NHS sites based on Wales. The sample size was determined by the clinical team based on their experience of running similar interventions to balance having enough participants to enable group discussion and shared experience, while keeping the group small enough to provide support and allow time for each individual to share their thoughts and experiences. (Tulip et al., 2020; Wilkie et al., 2021).

#### Inclusion criteria

- Confirmed diagnosis of ABI
- Ability to actively engage in the intervention as determined by their neuropsychological assessment scores and their treating clinician
- Living in the community
- Age 18 years or older
- Living within the catchment area of one of the participating health boards
- At least 3 months post injury at the point of recruitment, allowing time for spontaneous recovery and for the person to develop an awareness of their difficulties and the implications of this on their lives

#### Exclusion criteria

- Receptive or expressive language difficulties or extremely low memory function that may preclude people from engaging meaningfully
- Medical or psychosocial reasons (based on risk assessment by the referring clinician)
- Potentially disruptive to other group members, as determined by their treating clinician
- Not able to provide informed consent

Mentors were subject to the same criteria and additional criteria, judged by their treating clinician, related to their role as mentors, such as interpersonal skills and the ability to respond sensitively to others’ needs. All participants provided written informed consent to be included within the trial.

Of the 50 patients recruited, 23 were allocated to the PP intervention group and invited to be part of the qualitative focus groups. Twenty attended the focus groups and were included in this qualitative study. As shown in Table 1, participants were aged between 22 and 64 years and had a range of injury types and time since injury, from 1 year up to 11.7 years.

**Table 1.** Participant characteristics.

	n	%
**Injury type**		
Stroke	1	5%
TBI	6	30%
Extracerebral haemorrhage	4	20%
Brain Tumour	2	10%
Vascular malformations	2	10%
Hypoxia	2	10%
Other	3	15%
**Sex**		
Male	9	45%
Female	11	55%
**Age (years)**		
Median	46	n/a
Range	22 to 64	n/a
**Time since injury (years)**		
Median	3.1	n/a
Range	1.2 to 11.7	n/a
**Occupational status**		
Paid/self employed	8	40%
Unemployed	12	60%
Other^a^	0	0%

^a^ Includes students, retired and voluntary employment.

## Procedure

This study was part of an RCT feasibility study that adhered to the procedure as described in its published protocol (Fisher et al., 2024). Participants allocated to the intervention group and were invited to focus groups at the end of the final session of the intervention for qualitative analysis.

### Intervention

**Figure 1.**
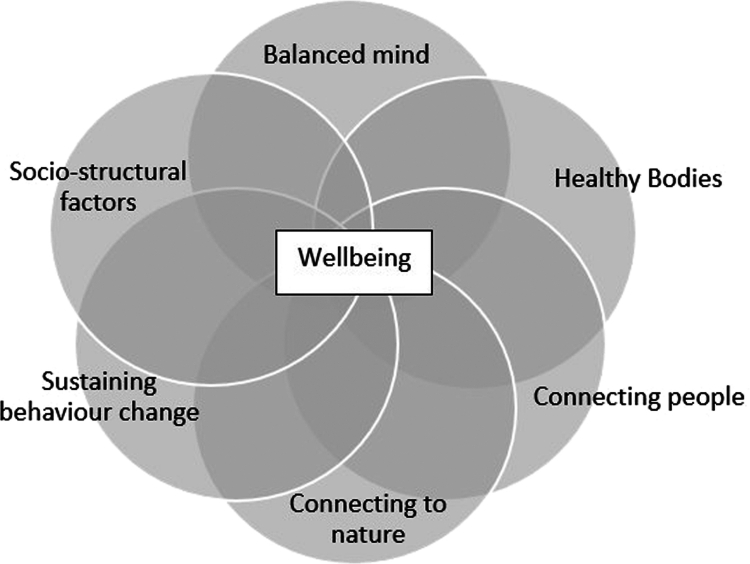
Summary of core theoretical components underpinning GENIAL well-being framework (Kemp & Fisher, 2022).

The PP intervention was developed collaboratively by academic and clinical psychologists, enhancing a previously delivered intervention (Tulip et al., 2020), with insights from participant experience and the latest developments in well-being theory (Kemp & Fisher, 2022; Mead et al., 2021; Tulip et al., 2020). Co-facilitated by two clinicians and two mentors, a semi-structured format was adopted, blending psychoeducation, practical well-being activities, and participant discussions. Course content was developed through the lens of the GENIAL framework (see Figure 1) (Kemp & Fisher, 2017b; Wilkie et al., 2021), integrating well-being theories such as PERMA (Seligman, 2011), psychological well-being (Ryff & Keyes, 1995) and subjective well-being (Diener, 1984) and drawing on developments in the field, including second-wave PP (Wong, 2011), which emphasises the utility of negative emotions as well as third-wave PP, which embraces multiple levels of scale focused on connection to self, others, and nature (Lomas et al., 2021; Mead et al., 2021). Content and activities were tailored to people living with ABI through clinical expertise and experience with prior interventions. Facilitators encouraged group discussions, providing opportunities for participants to connect and share their experiences of ABI with mentor encouragement by contributing their own experience. Sessions at each site lasted approximately two hours, with a long break midway through and additional breaks as needed (Table 2).

**Table 2.** Description of the 8-week positive psychotherapy group intervention (Fisher et al., 2024).

Session name (No.)	Summary of session content
(1) **Living with difficult emotions**	Difficult emotions are understandable following brain injury A series of exercises encourage participants to accept such emotions Participants learn coping skills, e.g., a ‘defusion exercise’ Also includes focus on mindful breathing exercises and self-compassion Difficult emotions motivate reflection and/or need for helpful changes ‘Snakes and ladders’ adaptation helps to make content memorable
(2) **Identifying & living using character strengths**	Discussion of the nature of character strengths Participants discuss results from a character strengths questionnaire Discussion of how participants might use their strengths Positive impacts of character strengths use are emphasised Positive self-statements ‘Snakes and ladders’ framework used to reinforce relevant concepts
(3) **Building positive emotions**	Positive emotions have psychological and health benefits They also help to cope with stress and adversity ‘Getting to Know Your Lemon’ mindfulness exercise Discussion of psychological flow, learned optimism and gratitude Activities include mindful eating exercise and ‘three good things’ activity Participants complete meaning and values clarification exercise Snakes and ladders’ framework used to reinforce relevant concepts
(4) **Connection between body and mind**	Participants learn about the mechanisms of the mind–body connection Focus on positive health behaviours, e.g., healthy diet, sleep, exercise Regulatory role of the vagus nerve, indexed by heart rate variability (HRV) Other techniques, e.g., singing, meditation, cold showers improve HRV Participants explore acute impacts of different activities on own HRV Highlights need for self-care, contextualised by healthy mind–body connection
(5) **Connection to others and the natural environment**	Focuses on connecting to others and nature, and links to health and well-being Science behind these connections, and how to improve well-being Responding to good news and events, e.g., active-constructive responding Variety of gratitude exercises, loving kindness meditation, volunteering Nature-based activities good for well-being (e.g., gardening) Also good for the environment (e.g., pro-environmental behaviours) Participants complete a ‘photo-journalist exercise’
(6) **Meaning & purpose**	Meaning and purpose give us a sense of direction and motivation in life May be enhanced and facilitated by focus on the self, collective and planet Discussion of photos representing areas of meaning for participants Areas of meaning linked to personal values as per exercise in Session 3 Behaviour-intention gaps constrain the translation of values into action Reflection on how those gaps might be overcome
(7) **Translating values into action**	Recap on participant’s strengths, values and important areas of meaning Explore extent to which participants are living a values-based life Participants identify and share areas where they are acting out their values Areas where they could better connect with their values are explored Participants set goals that support them to reconnect with some of their values Thoughts and feelings serve as barriers/facilitators to acting out values Return to the metaphor of ‘snakes and ladders
(8) **Behaviour change and managing ups and downs**	Focus on sustainable behaviour change Participants refine goals identified in Session 7 Challenges encountered when moving toward well-being are revisited Recap on strategies to manage those challenges Techniques and strategies that can be practised to support well-being reviewed Game of ‘snakes and ladders’ played to reinforce challenges and opportunities Variety of resources provided to help practice different techniques

### Focus groups

Eighteen participants attended focus groups from across the three NHS sites at the end of the final session, supplemented by two individual interviews for those who could not attend but wanted to give feedback. All interviews were conducted by the Research Trial Co-Ordinator (RTC), an experienced female researcher with training in psychology, who had established relationships with participants through trial co-ordination. Before starting, the participants provided signed and verbal consent for audio recording. Focus groups and interviews followed a similar semi-structured pattern, with open-ended questions covering study feasibility and exploring the impact on well-being (Appendix 1). The semi-structured nature enabled tailoring questions to fit the development of group discussion, enabling the interviewer to seek alternate and confirmatory views from participants and invite quieter participants to engage. Audio data, totalling 3 h 48 min, were transcribed orthographically, incorporating pauses and utterances to capture essence, and were anonymised.

### Analytic process

The first author, KD, began by reading the transcripts along with the audio recordings to familiarise themselves with the data. Reflexive Thematic Analysis (RTA) was conducted according to Braun and Clarke (2021). The flexibility of RTA enabled coding of data to be tailored to the type of experience shared; for example, commentary relating to feasibility, for example practical matters like travel, were coded semantically, whereas reflections linked to well-being, such as self-identity, were coded latently (Braun, 2022). Similarly, coding was both inductive, deriving meaning from what participants shared, and deductive, with meaning derived from linking the data to well-being theory (Braun, 2022). To supplement written notes and ideas and bring structure to the coding process, ATLAS.TI was used, which enabled visualisation of the codes within candidate themes, grouped initially based on similar content, and moved around as themes evolved throughout the analytical and writing process. As part of the reflexive process, candidate themes and names were presented in the form of a thematic map showing interrelations and key quotations for discussion with the research team who contributed their insight and expertise to shape the final themes.

## Analysis

**Figure 2.**
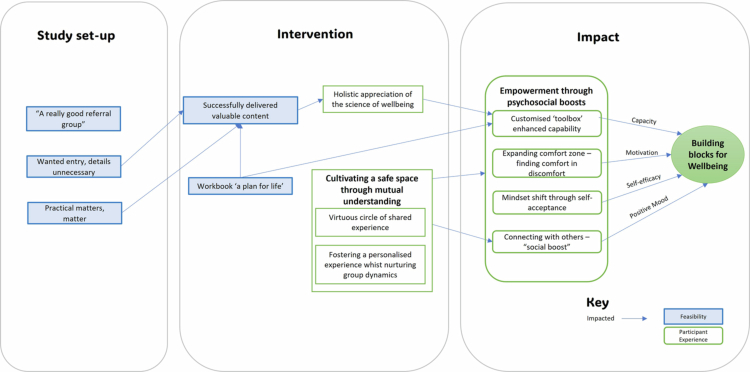
Thematic map demonstrating the interrelation between the feasibility and participant experience themes across the study process.

As presented in this thematic map (Figure 2), five themes captured the first study aim, to understand participants’ perspectives on study feasibility, and three themes illustrated the second aim, exploring participants’ experience of the intervention in building well-being.

### Feasibility

**Figure 3.**
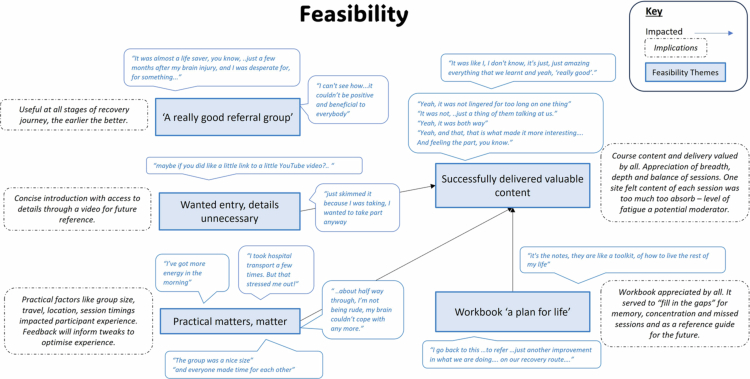
Thematic map presenting the feasibility themes, their implications for future studies and key illustrative participant quotations.

The thematic map presented in Figure 3 shows the relationship between the five themes across the study process, setup, and intervention. Themes are exemplified by key quotes and implications, which are expanded in the following analysis.

*Theme 1 — ‘A really good referral group’*

There was consensus that the intervention was beneficial irrespective of the time since ABI, which ranged from 3 months to 11 years. This contrasted with the experience from an earlier iteration of the intervention, where one participant felt too far along their journey to appreciate its benefits, potentially reflecting the value of the current iteration of the intervention, noting that this may also be a point of individual difference (Tulip et al., 2020). Participants commonly emphasised the value of attending earlier in recovery describing how early engagement could help to establish new routines following medical discharge (Hawley et al., 2022).

*“I think that's a ‘really good ‘referral group… when you're. really are confused and sad…. if you can get that connection at that point, it's going to be really, helpful.” (P1)*

However, several participants also noted that readiness to engage varied, with some expressing that they might not have felt comfortable participating immediately after injury. This highlights the importance of balancing early access with individual readiness to engage in a group setting. Research reinforces this interpretation, suggesting that peer support is most effective when introduced early in recovery, but only when individuals feel emotionally prepared to participate (Kersten et al., 2018).

*“I wouldn’t have done this a year …ago,* *…because we’re sat in a circle…uncomfortable, …but…it has benefitted me…” (P7)*

*Theme 2—Wanted entry, details unnecessary*

Feedback reflected the participants’ gratitude and eagerness to take part without fully disgesting the introductory material. While this did not reflect negatively on participant experience (to the contrary with some sharing how the introduction could have “sold” it better) it did leave gaps in their expectations of the study process and intervention.

*“…the introduction could have sold it more because it was way more beneficial than I thought” (P3)*

*“I think just a bit more about the group and how it was going to function” (P8)*

Unsuprisingly, given the prevelence of difficulties with attention and working memory after ABI, those that did review the participant information highlighted challenges absorbing it due to its length and written format, suggesting a “youtube video” as an alternative (Wood & Worthington, 2017).

*Theme 3— Practical matters, matter*

This theme captured practical arrangements such as travel, parking arrangements, location, group size, and session timings. In accordance with previous interventions, participants signalled the importance of getting these factors right, enabling participation, minimising fatigue, and maximising comfort (Gibbs et al., 2022; Payne et al., 2020; Tulip et al., 2020).

*“I wasn’t told, they didn’t say it was paid or not …it was sort of stressful and anxious.…” (P11)*

Participants were largely content with group size, which ranged from 8 to 10, was in line with comparable group interventions, and enabled space for everyone to contribute (Kotzur et al., 2023; Salas et al., 2021).

Useful insight was provided into what ‘right’ might look like in morning sessions, straightforward travel arrangements, ample breaks, and a welcoming non-medical setting (Kotzur et al., 2023).

*“It doesn’t feel like a hospital!. it’s a great unit!. so nice, friendly.” (P1)*

Travel played a critical role in the participant experience. Those in rural sites, spanning a large geographical area, experienced longer travel distances and pronounced fatigue (Cooper et al., 2009).

*“and its tough… today I’ve been up since before 7.00 am. driven from X to here…” (P15)*

*Theme 4— Successfully delivered valuable content*

There was undisputed appreciation for the quality of the course across all three sites, with variations in the aspects that were most beneficial.

*“yeah… knowing that everything that we did was really ‘well researched” (P5)*

The balance of the sessions, which ensured ample time for sharing experiences, and the varied holistic nature of the taught content, emphasising the’ why, supported engaged learning. This feedback was more pronounced at one of the sites whose facilitators had considerable experience delivering similar interventions.

*“* *… this was all connected and everything seemed to make sense” (P1)*

Some participants, specifically those in rural areas that experienced significant fatigue, felt that individual sessions were too detailed and recommended shorter sessions or more breaks.

Participants valued the key role clinicians played in supporting group dynamics, which is recognised, along with quality of content, as a key differentiator in successful group programmes (Kotzur et al., 2023).

*“she was like the glue that was holding us all together!” (P1)*

Some individuals noted specific improvements that would have enhanced their experience, such as more time for introductions, supporting individuals to speak up, and clarifying mentor roles, all of which can be addressed through updates to clinician manuals.

*Theme 5—Workbook ‘a plan for life’*

There was universal agreement regarding the value of the workbook in facilitating the intervention, which served as a reference manual for learning longevity and provided a ‘plan of action’ for participants to take forward.

*“it's the notes, they are like a toolkit, of how to live the rest of my life” (P5)*

It plugged gaps in missed sessions and gaps created by common challenges faced by those with ABI, such as poor memory (Hawley et al., 2022; Wood & Worthington, 2017).

*“You can always look back, or even when you have attended, because I forget,....now we got the information booklet, at the end I can always….look back!” (P2)*

#### Participant experience

**Figure 4.**
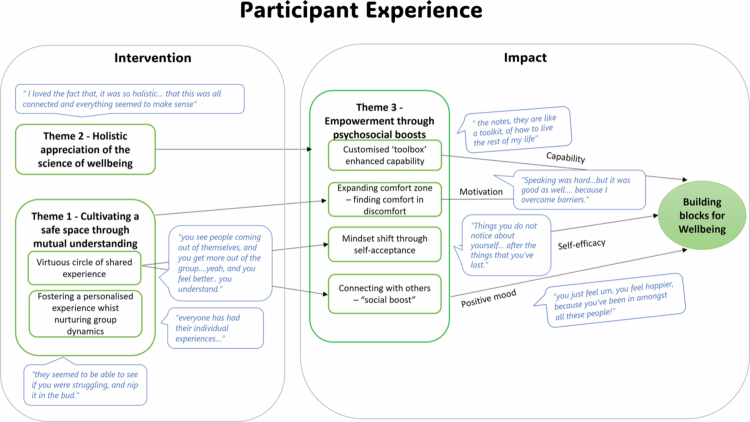
Thematic map, with key participant quotations, demonstrating the relationship between the themes and their contribution to building well-being.

As illustrated in Figure 4, themes related to the intervention itself, such as its holistic nature and the safe group-based therapeutic milieu, facilitated ‘empowerment through psychosocial boosts’ and contributed to building participant well-being.

*Theme 1—Cultivating a safe space through mutual understanding*

**Figure 5.**
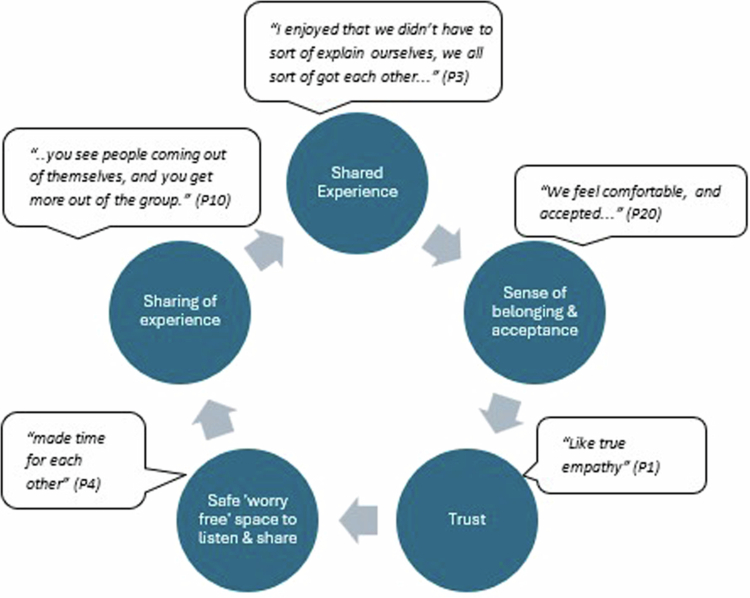
Visual representation of the virtuous cycle of shared experience with key quotations.

This theme captures the positive impact of a safe, nurturing learning environment on participant experience, reinforcing its criticality for successful rehabilitation in group settings (Salas et al., 2021). There were two critical components: participant shared experience (a) and effective facilitation of group dynamics (b) (Figure 5).

(a) **Virtuous circle of shared experience**

Sameness, through shared experience, fostered an immediate connection between participants (Salas et al., 2018), creating a sense of belonging (Kersten et al., 2018; Salas et al., 2021) and acceptance free from judgement, which established trust (Lau et al., 2021; Lyon et al., 2021).

Trusting one another to 'actually listen' with 'true empathy' made them feel able to share, promoting a safe peer-to-peer relational environment that acted as a catalyst for learning (Lau et al., 2021; Salas et al., 2018).

Shared experience constructed a picture of normality in the context of living with ABI, laying foundations for self-acceptance (Salas et al., 2018) and providing respite from the ‘loneliness’ and misunderstanding (Kotzur et al., 2023; Salas et al., 2021; Tulip et al., 2020).

*“I find myself pretending that I'm fine a lot. being able to just speak naturally in this group was. so, well, relaxing….” (P5)*

The mentors contributed their own lived experience, a “step ahead” in the journey, building a bridge of shared experience with group facilitation (Kersten et al., 2018).

(b) ***Fostering a personalised experience whilst nurturing group dynamics***

The facilitators effectively balanced support and encouragement, leaving participants feeling the benefits of a personalised experience within a group setting, which is considered key for successful rehabilitation (Kotzur et al., 2023).

*“personalisation was the big thing ….it did seem, you know, almost like it's tailored to yourself.” (P5)*

Consistent with other co-facilitated interventions, participants valued contributions from mentors who fostered a sense of normality while also offering support and encouragement (Kotzur et al., 2023; Salas et al., 2021).

*“I really felt that they opened us up a little bit” (P5)*

Facilitators’ nurturing approach, spending time with participants individually, and addressing unspoken needs fostered a space where participants felt able to contribute (Kotzur et al., 2023).

*“they seemed to be able to see if you were struggling, and nip it in the bud.” (P14)*

That said, individual reflections showed that it was not always possible to attend to each and every need, which may have been particularly challenging given the nature of the intervention needed to balance teaching psychoeducation while managing group dynamics.

*“helpful sometimes if maybe, they had asked to go around the group or noticed that so and so… hasn’t said anything.” (P8)*

***Theme 2***—***Holistic appreciation of the science of well-being***

The holistic well-being content (Gibbs et al., 2022; Kemp & Fisher, 2022) and nature and depth of explanation resulted in participants feeling respected as capable learners.

Participants appreciated how the intervention’s holistic content helped them understand the connection between multiple facets of well-being (Kemp & Fisher, 2022; Mead et al., 2021) enabling them to view well-being as something to aspire to despite ABI (Anderson, 1995; Ryff et al., 2006).

*“* *… I found, rather than just being told to do it because it's good for you. You ‘actually’ learn ‘why’ and ‘how’, it's the mind and the body connected.” (P5)*

The combination of teaching why, what, and how meant that participants bought in to what they learned, helping them to “believe it” This approach left them feeling empowered to put the theory into practice and share it with others (Hertwig & Grüne-Yanoff, 2017).

*“…we believe it will relax us, this will ‘calm’ us down. In the group they went into just enough science about why we're doing it, and the biological effect this mental work has on us.” (P5)*

This laid the foundation for psychological boosting, specifically training individuals with the knowledge needed to enhance their capabilities (Fabian & Pykett, 2022; Hertwig & Grüne-Yanoff, 2017; Kemp & Fisher, 2022).

*“actually being explained. what this will do to you, physiologically, why it's beneficial. actually got me practising that stuff …” (P5).*

***Theme 3***—***Empowerment through psychosocial boostsing***

Participants reported feeling “boosted” with improved mood and a sense of empowerment, equipped with the capability and mindset to “look forward” and “make the most of life”. This exemplifies the concept of psychological boosting, whereby people are trusted and conciously trained with tools to make their own decisions (Fabian & Pykett, 2022; Hertwig & Grüne-Yanoff, 2017). Four sub-themes reflecting the output of the components of psychosocial boosting made up this theme: (a) capability, (b) motivation, (c) self-efficacy, and (d) social connection.

(a) ***Customised ‘toolbox’ for enhanced capability***

Through learning and practice, participants customised their own “toolbox” of skills, providing them with the knowledge to improve their own well-being, in line with aims of comprehensive rehabilitation programmes (Salas et al., 2021) and in contrast to the ‘find and fix’ medical model (Anderson, 1995).

*“coming here has given me the tools on how to cope and how and what to say and everything” (P16)*

Participants reflected on how they had learned to navigate difficult emotions and savour positive emotions, core aspects of well-being theories, which improved their mood and personal relationships (Diener, 1984; Seligman, 2011).

*“it’s helped me with my relationship as well because … now I've calmed down. I know how to deal with it and not shout.” (P2)*

Participants shared, with pride, how they had used their customised “toolbox” to set goals and resolve challenges in different settings, inspiring them to put what they had learned into “action” and exemplifying a sustained ‘psychological boost’ (Fabian & Pykett, 2022; Hertwig & Grüne-Yanoff, 2017).

*“But now, when I come up against a, a barrier or an obstacle… I know how to deal with it” (P17)*

*“I leave here feeling inspired… ‘Like, right, what have I learned today that I can put into action?” (P18)*

(b) ***Expanding comfort zone—finding comfort in discomfort***

The positive environment fostered by the group resulted in the participants pushing themselves out of their comfort zone to connect, share, and try new things.

*“Speaking was hard… but it was good… because I overcame barriers” (P6)*

This practice built resilience, positively linked to well-being outcomes for people with ABI (Bertisch et al., 2014), which served to expand their comfort zone (Salas et al., 2021).

*“So, you're going outside of your comfort, your comfort zone, but in a good way…” (P4)*

In much the same way that their ‘toolbox’ upskilled capability; expanding their comfort zone left participants feeling intrisincally motivated to take on more inside and outside the group (Fabian & Pykett, 2022; Heyman & Dweck, 1992).

*“I’d leave here. sometimes tired… but always feeling … hope… and almost ready to take on anything… reached out to friends. getting more exercise. it's affected so much of my life, I just feel more positive about everything” (P5)*

(c) ***Mindset shift through self-acceptance***

Through group interactions, participants compared and contrasted their experiences, enabling them to embrace “different aspects” of themselves (Festinger, 1954; Lyon et al., 2021).

This shifted them to see beyond “the things lost” and begin to accept themselves for who they are now (Lyon et al., 2021).

*“I can still do things and you know I can ‘actually see’ that I can’t do some things” (P2)*

*“yeah… it’s accepting, it’s coming to that acceptance” (P1)*

Adjusting to changes in self-identity is a common challenge (Vaghela et al., 2023) with a positive shift in self-perception, which is considered an early enabling stage of recovery (Gracey et al., 2009).

*“I can see everybody at different stages right where I was at that stage, and they helped me get over that hurdle, and then I, I faced another hurdle, but then everybody in the group is, is coming up behind me, like a horse race! … I can see how far I've come and how far everybody else in the group has come because of this this course.” (P7)*

As illustrated by this quotation, comparison with others can help boost self-esteem through appreciation of where they are in relation to others, leading to self-efficacy being a core component of psychological boosting (Fabian & Pykett, 2022; Festinger, 1954).

*“I feel that the most I've changed, is my, my outlook. … I got loads from looking around the room and seeing how other people have just really progressed through it.” (P5)*

Social comparison contributed to a positive future outlook, accepting it “may be a different future” than envisaged, evidencing increased life satisfaction associated with post-traumatic growth (Bellon et al., 2022; Diener, 1984). Consistent with Ryff & Keyes, (1995) psychological well-being theory, their language demonstrated autonomy for “making the most of” their own life and self-efficacy, a belief in their ability to succeed, considered to be key facilitators in succesful rehabilitation (Nott et al., 2021).

(d) ***Connecting with others—“social boost”***

Aligning with experience from peer support groups, learning while connecting boosted participants’ mood, which continued beyond the sessions, consistent with the critical role of social interaction in building well-being (Kemp & Fisher, 2022; Salas et al., 2021).

“*I was running a lot better moods throughout the week” (P3)*

*“it's changed a lot.I can work for longer. I could just go on and upwards” (P5)*

Consistent with findings from peer-support interventions, participants attached meaning to the support they provided one another (Aterman et al., 2023) demonstrating that they were starting to extend their practice of well-being beyond themselves, supporting the bidirectional relationship of well-being of self and others (Corral-Verdugo et al., 2021; Fisher et al., 2019).

*“it was nice for us to hear you, and to listen and to offer our support to you...., it's nice giving that to each other” (P3)*

Participants recognised the value of their relationships wanting to sustain connection and feeling “sad” about it ending. One group felt an attachment to the location where they had built connections, alinging with the concept of ‘social boosts’ whereby participants co-created a community to connect and reduce social isolation (Fabian & Pykett, 2022).

*“Maybe...for a coffee… just sitting at the table....back then,....that was just lovely for me” (P3)*

## General discussion & conclusion

Overall, this study found that the intervention was both feasible and well-received, with participants valuing its holistic approach, structured psychoeducation, and the integration of well-being science. These insights provide valuable guidance for scaling and future applications. The findings support the feasibility of a full-scale RCT and demonstrate that well-being can be actively promoted and improved in individuals with ABI.

Participant experience demonstrated the feasibility of delivering this psychosocial group intervention and its effectiveness in enhancing and sustaining well-being through psychosocial boosting in people with ABI (Fabian & Pykett, 2022). Participants demonstrated improvements across multiple facets of well-being such as improved mood, hope, and resilience (Seligman, 2011) exemplifying flourishing (Ryff & Singer, 2008). This added to the literature further evidencing the possibility of building well-being in people with ABI despite its chronic nature (Bellon et al., 2022). The intervention blended psychoeducation, grounded in second- and third-wave positive psychology and drawing on systems theory to understand connection as dynamic and interdependent, with peer mentoring in a group format. This approach advanced earlier PP work with people with ABI (Cullen et al., 2018; Kemp & Fisher, 2022; Tulip et al., 2020; Whiting et al., 2020) by applying a holistic model of well-being that integrates connection to self, others, and the natural environment across three sites.

In contrast to criticism about the failure to translate research into practice (Grimshaw et al., 2012), this intervention was specifically developed to address clinical needs and enhanced with both developments in wellbeing theory and participant insights from prior research (Tulip et al., 2020). Compared with the earlier iteration described by Tulip et al., the current version reflects a broader theoretical foundation that moves beyond a primarily PP focus toward a systems-informed approach. The GENIAL well-being framework (Kemp & Fisher, 2022) underpinned this evolution, incorporating principles from second- and third-wave PP and framing connection to self, others, and nature as dynamic and interdependent. These refinements were shaped not only by theoretical advances but also by feedback from participants and peer mentors, whose experiences helped tailor content to be more relevant, accessible, and personally meaningful. Refinements were also shaped by feedback from participants and peer mentors, whose experiences helped tailor content to be more relevant, accessible, and personally meaningful. This aligns with principles underpinning best-practice healthcare service delivery in Wales focused on value-based healthcare, delivering outcomes that matter to patients (Government, 2022a, 2022b). Participants’ reflections evidenced that this content, together with the way it was taught, explained scientific connections and interconnections between the facets of well-being, resulting in a depth of understanding that left them feeling empowered to put it into practice, exemplifying psychological boosting (Fabian & Pykett, 2022; Kemp & Fisher, 2022; Mead et al., 2021).

Participant reflections predominantly highlighted the value of connection with others in supporting wellbeing throughout the course. For many, this connection was not simply a topic of discussion but an immersive experience; the shared group setting itself fostered belonging, mutual understanding, and social confidence, key mechanisms in psychological recovery following ABI. While fewer references were made to connection with nature, this likely reflects the emphasis on social and emotional rehabilitation during this stage of recovery. While this intervention’s core aim was to promote individual well-being (Rosset et al., 2024), participant experience demonstrated enhanced well-being by supporting others. This evidenced the bi-directional link between well-being of self and others (Hui et al., 2020; Payne et al., 2020) and their eagerness to continue supporting one another exemplified a social boost (Fabian & Pykett, 2022). These insights indicate the potential for holistic psychosocial interventions to empower people with ABI to optimise their own well-being, addressing unmet psychological and social needs (Fabian & Pykett, 2022; Odumuyiwa et al., 2019). However, the intervention is grounded in a systems-informed view of connection, linking self, others, and nature as interdependent facets of wellbeing (Kemp & Fisher, 2022). Activities promoting self-care, character strengths, and embodied awareness supported connection to self, which in turn laid the foundations for meaningful relationships with others and the natural environment. This relational approach aligns with Tulip et al. (2020) recommendation to enhance future iterations by deepening opportunities for community and nature connection. Building on this, future delivery could further strengthen the nature-connection component, for example, by embedding structured outdoor activities, reflective exercises, or nature-based metaphors that reinforce the reciprocal relationship between personal wellbeing and the natural world. Such developments would extend the framework’s systemic emphasis on relationality and sustainability, reflecting emerging evidence for the restorative and integrative benefits of nature connection in neurorehabilitation contexts (Gibbs et al., 2025; Richardson & McEwan, 2018).

Successfully scaling the intervention as a referral group, in line with participants’ recommendations, has the potential to improve individual outcomes more broadly, consistent with value-based service delivery (Government, 2022a; Norman et al., 2023). Participants provided valuable, constructive feedback on enhancing the patient experience when scaling. For example, they suggested improving accessibility of introductory material through videos and making practical consideration considerations such as travel, location, and session timing as straightforward as possible. In regional locations, more local provision would help reduce travel and fatigue, although this may not always be possible due to participant numbers and clinician resources. Other mechanisms for delivery could be considered, such as online or hybrid approaches, to reduce the travel burden on participants. Whilst this intervention was developed directly in response to patient demand and has been improved informally through patient feedback, going forward it would be valuable to embed patients more fully in co-designing improvements as we spread and scale. Building on the insights gathered in this study, involving patients in developing introductory videos, refining client manuals, and shaping practical delivery approaches are key ways to enhance the intervention and ensure it is patient-led, thereby improving the future patient experience.

While qualitative insights, capturing participants’ perspectives, are useful in shaping future research (O’Cathain et al., 2015), to enable translation into wider practice, stronger research evidence is needed to substantiate promising indications (Gimigliano & Negrini, 2017). Supplementing the qualitative participant insights captured in this study with quantitative measures of well-being would generate a more nuanced understanding of the intervention’s impact on participant well-being. Though conducted across multiple sites, with only one group per site, participant numbers were low. Extending to more sites and groups, coupled with collecting quantitative data to supplement qualitatie data would strengthen research evidence of its effectiveness. Furthermore, while participant reflections indicated an intention to continue building their own well-being, suggesting longevity, an anticipated impact of psychological boosting, this remained untested with focus groups conducted immediately after the intervention itself. Focus groups using ripple effects mapping, with a follow-up period post-intervention, would provide an understanding of the extent and sustainability of the intervention in building well-being over time (Gibbs et al., 2025; Nobles et al., 2022).

Insights from this qualitative study indicated the potential for holistic group-based PP interventions, co-created through participant reflections, to empower people with ABI with the capability, motivation, and self-efficacy to master their well-being. With psychological needs commonly cited as unmet in people with ABI, there is scope for interventions such as this to be scaled and implemented as referral groups to enable people to tailor themselves to meet their own needs. Participant insights indicated the feasibility of scaling this to a full mixed-methods RCT, which would pave the way for this PP intervention to be put into practice to deliver better outcomes for people with ABI on a broader basis.

## Supplementary Material

Supplementary Material Appendix.

## Data Availability

The data that support the results of this study are ethically restricted and are not openly available within the public domain. This is because a majority of the transcripts contain potentially identifying contextual and sensitive patient information that could compromise research participant privacy if combined with local knowledge of the service and participants involved. These restrictions were imposed by the Research and Development Department at the Swansea Bay University Health Board (Room 104, Institute of Life Sciences 2, Swansea University, Singleton Campus, SA2 8PP; SBU.RandD@wales.nhs.uk). However, given that this local and contextual information is not known to outside researchers, access to anonymised transcripts may be granted to researchers upon reasonable request and with permission from the Research and Development Department. Please contact the Team Co-ordinator at the Regional Neuropsychology and Community Brain Injury Service, Swansea Bay University Health Board, Morriston Hospital, Morriston, Swansea, SA6 6NL. Data requests should be directed to sbu.communityneurorehabilitation@wales.nhs.uk and reviewed on a case-by-case basis.

## Trial registration

ISRCT N1269 0685, registered with the ISRCTN, the UK’s Clinical Study Registry, on November 11, 2020, available at https://doi.org/10.1186/ISRCTN12690685.
