# Supplementary material for: Empowering people with acquired brain injury to master their well-being: a thematic analysis of participant experience of an 8-week positive psychotherapy group
Source: Int J Qual Stud Health Well-being. 2025 Dec 10;20(1):2595847. doi: 10.1080/17482631.2025.2595847 (PMC12699746; doi:10.1080/17482631.2025.2595847)
Supplement: Supplementary Material — Appendix. [file ZQHW_A_2595847_SM3412.docx]

**Appendices**

Appendix 1: Semi-structured focus group topic guide

***Note for interviewer: Thank participant for agreeing to share their experiences. Make sure they are comfortable and aware of right to withdraw. Reiterate confidentiality policy and ask if they have any questions before the discussion begins. Ensure participant is aware that we are keen to understand their unique experiences and that there is no right or wrong answer.***

*“Thank you for agreeing to share your experiences with us. We’re keen to understand how you felt about the group and appreciate you taking the time to voice your experiences”.*

*“The discussion may take a little while in all, so if at any point you feel you need to take a break or you wish to withdraw from taking part, please just let me know. The information you provide will be kept strictly confidential such that outside readers will not be able to identify you from the product of this discussion”.*

*“An audio recording will be made of this interview. During the recording please do not give any information that will identify you or other group members. If a group member accidentally gives some information which will identify themselves or another group member, it will be removed from the transcript of the audio recording. The audio recording will be kept on a secure NHS computer and it will be deleted as soon as it is typed up by a member of the research team.”*

1. **Could you briefly describe the Positive Psychology group that you attended?**
2. **Could you describe how you were recruited to take part in the group?**

- Could we improve upon how we did that?
- You were given some information before consenting to the research. How did you find the information provided?
- Was the research process clear to you when you consented to participate?
- Having completed the group sessions, do you think there is anything additional you would have liked to know before consenting to the research? Or should future participants be informed?

1. **Did you understand how the randomisation process worked, in terms of why some people were allocated to a group and why some people were not?**
2. **How did you get to the sessions?**

- Was it easy or difficult?
- Did you find the building/group location accessible?
- Was the group convenient for you?
- If you miss some sessions, is there anything that could be done to help you and others attend more sessions?

1. **What did you think about the data collection process**

- We understand that there were quite a few questionnaires to complete at appointments, both before and after the groups took place. How did you feel about them?
- How did you feel about the number of questionnaires to complete?
- How did you feel about having your HRV measured?
- Did you find any aspect of the information required from you unreasonable/unnecessary?
- Could we have done anything differently to improve the data collection process for you?

1. **Did you have any ideas of what may happen in the group?**

- Was there anything unexpected or surprising to you?
- Was there anything you felt uncomfortable with?
- What did you think about the role of the Mentors of your group?

1. **Could you describe a typical day where you attended the group?**

How did you feel about attending the group before the first session?

- How did you feel after the session?
- How did you feel about the group at the end?

1. **What did you think about the way the group was run?**

- How did you feel about the number of people in the group?
- Did you feel that you could talk to the group leader with any concern you had?
- Was there anything you would change?
- What did you think about the balance among presenting information, experiential exercises, and interactive discussions?
- Were the presentation slides easy to follow?
- What did you think about the length of the session?
- Did you feel confident that what you said in the group would remain confidential?

1. **What do you think you took away from the group?**

- Is there anything you have found particularly helpful or unhelpful?
- Has anything changed for you since going to the group?
- Could you give an example of how you use any of the skills that you have learned?
- Are there any skills or techniques that you have not tried? Why?
- Do you think there are any additional materials we could provide to help practice some of the techniques at home/in general?

1. **You were given a workbook summarising each session of the group as a memory aid during and after the group, did you find this useful nor not?**

- Would you change anything about it?
- Did you used the workbook in-between sessions?

1. **How would you sum up your experience of the group?**

Finish with:

*“We really appreciate you taking this time to share your experience. Is there anything else that you would like to add, or anything you think I should know to fully understand how you feel about the group?”*

Appendix 2: COREQ Checklist

| **Item No** | **Guide Questions/Description** | **Reported in Section** |
| --- | --- | --- |
| **Domain 1: Research team and reflexivity** |  |  |
| **Personal Characteristics** |  |  |
| 1. Interviewer/ facilitator | Which author/s conducted the interview or focus group? | Method – Procedure – Focus Groups |
| 2. Credentials | What were the researcher’s credentials? E.g., PhD, MD |  |
| 3. Occupation | What was their occupation at the time of the study? |  |
| 4. Gender | Was the researcher male or female? |  |
| 5. Experience and training | What experience or training did the researcher have? |  |
| **Relationship with participants** |  |  |
| 6. Relationship established | Was a relationship established prior to study commencement? | Method – Procedure – Focus Groups |
| 7. Participant knowledge of the interviewer | What did the participants know about the researcher? e.g. personal goals, reasons for doing the research? |  |
| 8. Interviewer characteristics | What characteristics were reported about the interviewer/facilitator? e.g. Bias, assumptions, reasons and interests in the research topic |  |
| **Domain 2: Study design** |  |  |
| **Theoretical framework** |  |  |
| 9. Methodological orientation and Theory | What methodological orientation was stated to underpin the study? e.g. grounded theory, discourse analysis, ethnography, phenomenology, content analysis | Method - Design |
| **Participant selection** |  |  |
| 10. Sampling | How were participants selected? e.g., purposive, convenience, consecutive, snowball | Method Participants |
| 11. Method of approach | How were participants approached? e.g., face-to-face, telephone, mail, email | Method Participants |
| 12. Sample size | How many participants were in the study? | Method Participants |
| 13. Non-participation Setting | How many people refused to participate or dropped out? Reasons? | N/A part of wider RCT feasibility study |
| 14. Setting of data collection | Where was the data collected? e.g., home, clinic, workplace | Method – Procedure – Focus Groups |
| 15. Presence of nonparticipants | Was anyone else present besides the participants and researchers? | Method – Procedure – Focus Groups |
| 16. Description of sample | What are the important characteristics of the sample? e.g. demographic data, date | Method Participants |
| **Data collection** |  |  |
| 17. Interview guide | Were questions, prompts, and guides provided by the authors? Was it pilot tested? | Appendix 1 |
| 18. Repeat interviews | Were repeat interviews carried out? If yes, how many? | N/A |
| 19. Audio/visual recording | Did the research use audio or visual recording to collect the data? | Method – Procedure – Focus Groups |
| 20. Field notes | Were field notes made during and/or after the interview or focus group? | N/A |
| 21. Duration | What was the duration of the interviews or focus group? | Method – Procedure – Focus Groups |
| 22. Data saturation | Was data saturation discussed? | N/A |
| 23. Transcripts returned | Were transcripts returned to participants for comment and/or correction? | No |
| **Domain 3: analysis and findings** |  |  |
| **Data analysis** |  |  |
| 24. Number of data coders | How many data coders coded the data? | Method – Procedure Analytic Process |
| 25. Description of the coding tree | Did the authors provide a description of the coding tree? | N/A |
| 26. Derivation of themes | Were themes identified in advance or derived from the data? | Method – Procedure Analytic Process |
| 27. Software | What software, if applicable, was used to manage the data? | Method – Procedure Analytic Process |
| 28. Participant checking | Did participants provide feedback on the findings? | No |
| **Reporting** |  |  |
| 29. Quotations presented | Were participant quotations presented to illustrate the themes/findings? Was each quotation identified? e.g., participant number | Analysis – Figure 3.; Figure 4 |
| 30. Data and findings consistent | Was there consistency between the data presented and the findings? | Analysis |
| 31. Clarity of major themes | Were major themes clearly presented in the findings? | Analysis – Figure 3.; Figure 4 |
| 32. Clarity of minor themes | Is there a description of diverse cases or a discussion of minor themes? | Analysis – Figure 3.; Figure 4 |
